# Seaweed functional strategies, functional groups, and taxon dynamics through a 213-year historical series of Rio De Janeiro Bay

**DOI:** 10.1038/s41598-024-77284-y

**Published:** 2024-10-29

**Authors:** João P. G. Machado, Vinícius P. Oliveira

**Affiliations:** 1 Institute of Biology, Federal University of Rio de Janeiro (UFRJ), Rio de Janeiro, Brazil; 2 Institute of Biology, State University of Rio de Janeiro (UERJ), Rio de Janeiro, Brazil

**Keywords:** Biodiversity loss, Functional form, Marine ecology, Marine vegetation, Morpho-functional group, Urban ecology, Biodiversity, Ecological modelling, Urban ecology, Marine biology

## Abstract

From Darwin’s visit to nowadays, we analyzed the changes in species- and genus-level richness and turnover, equity, diversity, composition, as well as functional group and functional strategy dynamics over a 213-year historical series of the seaweed assemblage from Rio de Janeiro Bay. We developed new analysis methods to serve as a protocol for worldwide functional and bioindicator assessment of seaweed assemblages. Over the two centuries of changes tracked, the integration of taxonomic, functional group, and functional strategy analyses unraveled a trajectory of seaweed assemblage restructuring under human disturbance. Our taxonomic analyses showed a high species and genus turnover and a decline in richness, with the local extinction of the *Sargassum* genus, responsible for warm temperate kelp forests in the Western Atlantic. Seaweed functional group data presented a trend of morphological simplification and miniaturization, with the decline of marine forest canopy and subcanopy-forming groups favoring filamentous and crustose algae dominance. Functional strategy data showed the historical decline of competitive structuring as an assemblage driver in favor of disturbance and stress-structuring, as highlighted by the competitive strategy decrease, coupled with stress-tolerant and ruderal strategies rise across metrics. These changes were successfully portrayed by the new ecological index we proposed, the C/SR index. It is the first quantitative index for seaweed assemblages, designed to be a trackable metric for conservation, monitoring, and restoration efforts.

**Supplementary Information:**

The online version contains supplementary material available at 10.1038/s41598-024-77284-y.

## Introduction

**Fig. 1:**
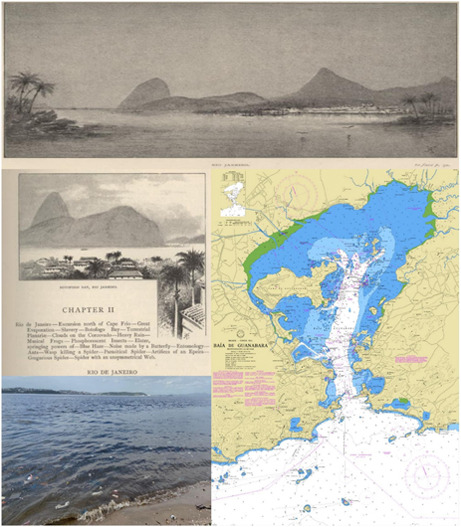
Rio de Janeiro Bay from Darwin’s Beagle voyage to nowadays. Clockwise: upper left and top: illustrations from Darwin’s Journal of Researches; right: modern nautical charter of Rio de Janeiro Bay; bottom left: a view from the modern shoreline.

Two centuries ago, when Darwin visited Rio de Janeiro Bay, then called Botafogo Bay and now Guanabara, he remarked on the breathtaking sight of tropical scenery, exuberant plants, and curious animal life^1^. What he saw then was an overall pristine environment, with a low degree of human impact on the marine environment. Ever since the Portuguese court fled from the Napoleonic invasion of Portugal, and due to it being the capital of the Brazilian Empire and Republic up to 1960, Rio de Janeiro experienced, from the 19th century onwards, increased urbanization, industrialization, and anthropization of its bay area^2–5^. Coastal alteration, pollution of the bay’s tributaries from untreated domestic and industrial sewage, oil spills, waste disposal along the bay’s margins, industrial contaminant discharge, heavy international maritime traffic, bottom trawling, and overfishing continue to leave their environmental mark on Rio de Janeiro’s Guanabara Bay today^5–10^ (Fig. 1).

As Rio de Janeiro Bay sits on the warm temperate belt, seaweeds are the main structuring factor of its rocky shores^5,11–13^. Being fixed to the substrate and showing direct responses to changing biotic and abiotic conditions, seaweeds offer a unique group to assess the functional changes over marine life across two centuries of increasing human disturbance^14–16^. The analysis of seaweed assemblages also allows the bias of phylogenetic autocorrelation to be minimized, as they display the greatest phylogenetic diversity of all multicellular organisms^17–19^.

However, as seaweed taxonomy remains unstable and not well-resolved^20^, it is problematic to track the changes brought about by anthropogenic pressures relying on taxonomic changes alone^21^. The functional ecology approach offers a way to tackle this shortcoming while also expanding the analysis to better model ecosystemic function and changes in community structure^22^. Functional analyses of 213-year changes to seaweed assemblages besides being necessary for local conservation and restoration efforts would also allow cross-comparison with other bays worldwide.

To uncover the 213-year historical series of functional changes over Rio de Janeiro Bay, we ask: (1) What changes have occurred across the seaweed taxa? (2) How have functional groups and strategies changed? (3) Has anthropization favored one evolutionary strategy over another? We also present and test our newly proposed ecological index, the C/SR ratio index, as a means of quantitatively tracking functional changes in rocky shores and reefs with seaweed dominance.

## Materials and methods

### Occurrence data

We extracted the occurrence record data for 245 seaweed species, from 77 genera of three phyla, by taxonomic occurrence data on Rio de Janeiro’s Guanabara Bay, from 1800 to 2013, following the most up-to-date taxonomic list database, based on published species occurrence data and herbarium material examination^5,6^. Occurrence data through time was divided into four periods: 1800–1927, 1940–1978, 1983–2006, and 2010–2013^5,6^. The first period provides the seaweed assemblage historical baseline under low anthropogenic pressure, followed by three periods of major human impact growth.

### Taxonomic analyses

We assessed taxonomic turnover across the assemblage’s 213-year time series by calculating changes in richness and turnover rate for species and genera. Genus-level analysis was undertaken due to the frequent revision of tropical species naming e.g.,^23–26^. Nomenclature at the genus level remains more stable and less prone to being invalidated and reviewed for tropical seaweeds overall^26^. Both species and genus richness per period were calculated by the sum of present species and genera in each period, respectively. Period by period changes in species and genera richness were assessed as richness turnover.

### Functional analyses

#### Functional group classification

Species were also classified using the Steneck and Dethier^27^ seaweed functional group model and Grime’s functional strategies^27–31^. Steneck and Dethier^27^ seaweed functional groups were originally derived from Grime’s functional strategies^27–29,31^. Thus, we reconverted the derived groups into the original strategies for the latter’s classification (Table 1).

**Table 1:** Functional group models were used.

Steneck and Dethier groups	Conversion	Grime strategies
Crustose algae	→	Stress-tolerant
Articulated calcareous algae	→	Stress-tolerant
Filamentous algae	→	Ruderal
Foliose algae	→	Ruderal
Corticated foliose algae	→	Ruderal
Corticated macrophytes	→	Competitive
Leathery macrophyte	→	Competitive

#### Functional dynamics

We assessed the functional dynamics of the seaweed assemblage by calculating the following metrics for each functional group and strategy: species richness, relative frequency, taxonomic importance per group and strategy (1 - relative frequency), composition changes (Jaccard dissimilarity index), functional equitability (Pielou’s Evenness Index applied for functional groups and strategies, $\:\frac{H{\prime\:}}{\text{l}\text{n}\left(G\right)}$), and functional diversity (Shannon diversity index adapted for functional groups and strategies, $\:-\sum\:_{i=1}^{n}({p}_{\text{i}}\bullet\:\text{l}\text{n}\left({p}_{\text{i}}\right))$ ).

### C/SR index: a quantitative index for primary producers

We formulated a mathematical model to evaluate functional change in seaweed assemblages. This model calculates the ratio of the relative frequency of competitive strategists (C) to the combined relative frequency of stress-tolerant (S) and ruderal strategists (R) **(**Eq. 1).

1 $${\textstyle\frac{\mathrm C}{\mathrm{SR}}}\;\mathrm{index}\;=\frac{\mathrm C}{\mathrm S+\mathrm R}\;$$

## Results

### Species and genus-level dynamics

**Fig. 2:**
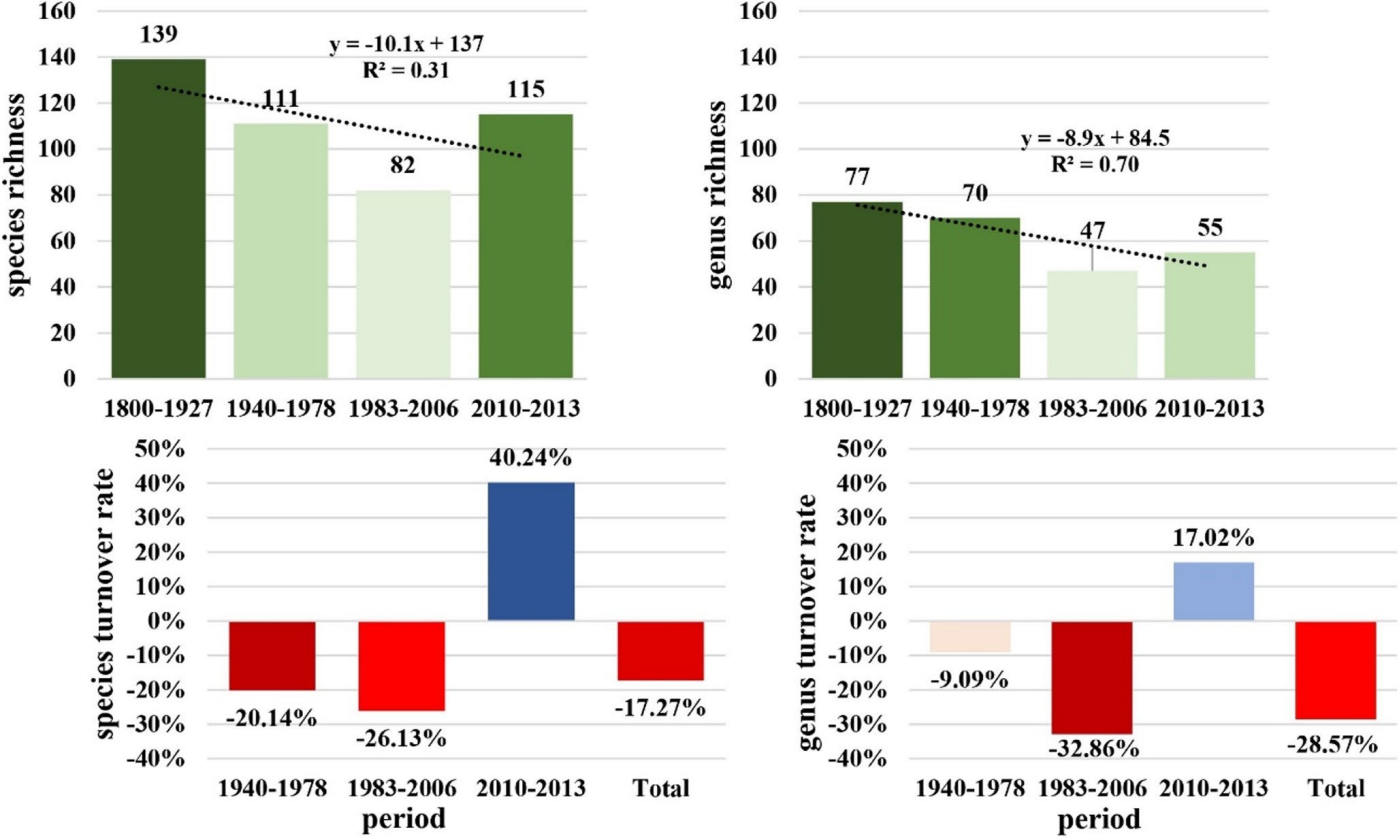
Species and genus richness and turnover rate across 213 years. Period by period changes were assessed as richness turnover.

Seaweed species richness in the Rio de Janeiro bay area showed a notable decline over two centuries (Fig. 2; Supplementary Table 1). Starting from a baseline of 139 species in 1800–1927, by 1940–1978, richness dropped to 111, with a 20% loss in species composition. This trend continued through 1983–2006, reaching a low of 82 species and a turnover rate of -26%. A reversal occurred between 2010 and 2013, with richness increasing to 115, reflecting a small recovery. However, the long-term average turnover shows a general decline in species richness. The total species turnover rate from 1800 to 2013 was − 17%.

Genus-level dynamics mirrored the species-level trends. From 1800 to 1927 to 1940–1978, genus richness declined modestly from 77 to 70. A larger drop occurred in 1983–2006, when richness fell to 47 genera, with a higher turnover rate of -33%. By 2010–2013, there was a modest recovery in genus richness, with it rising to 55 in total. The overall trend shows a gradual decrease in genus richness with a slight recent uptick. The total genus turnover rate from 1800 to 2013 was − 29%.

### Functional group dynamics

#### Species richness

**Fig. 3:**
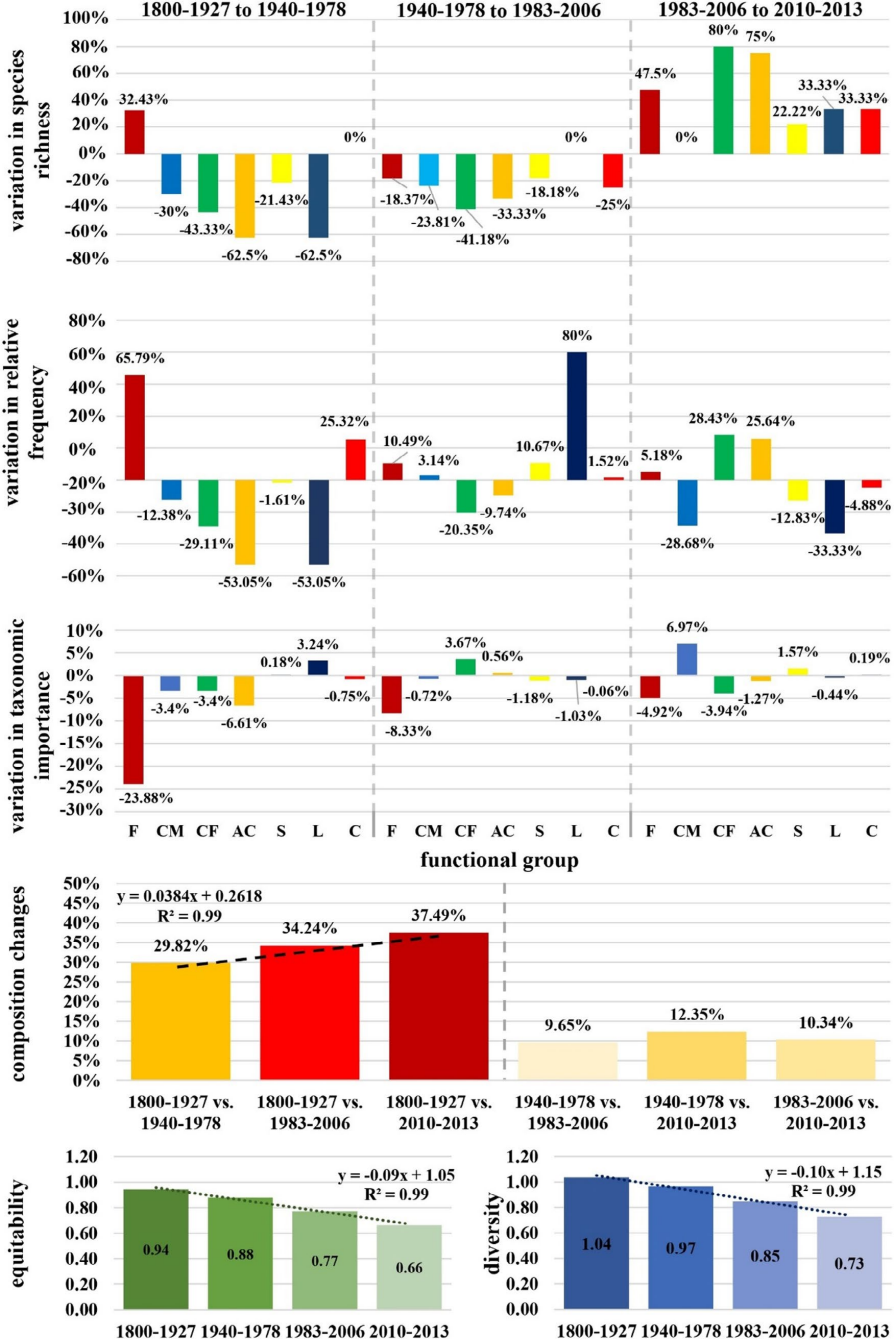
Seaweed functional group dynamics across 213 years of anthropogenic disturbance. Letters stand for seaweed functional groups. F, filamentous group. CM, corticated macrophyte group. CF, corticated foliose group. AC, articulated calcareous group. S, sheet (foliose) group. L, leathery macrophytes. C, crustose group.

Species richness within functional groups underwent major shifts over time (Fig. 3). Notably, the filamentous group saw an initial 32% increase in richness from the previous baseline, while the articulated calcareous group saw a steep decline of -63%, followed by similar reductions in the corticated foliose and leathery macrophyte groups by -43% and − 63%, respectively. The filamentous group’s species richness fluctuated in later periods, ultimately increasing by 48% between 1983 and 2006 and 2010–2013. The corticated foliose group rebounded strongly, with an 80% increase during this final period, signaling some recovery.

#### Relative frequency

Among the fluctuation in relative frequency, it is worth highlighting that the filamentous group saw the largest increase, of 66%, from 1800 to 1927 to 1940–1978, while the articulated calcareous and leathery macrophyte groups experienced the greatest declines at -53%. These trends continued, with the filamentous group’s frequency rising, while the corticated macrophyte group saw decreases, including a 29% drop by 2010–2013. The corticated foliose group notably recovered, increasing by 28% (Supplementary Fig. 1).

#### Taxonomic importance within functional groups

Notably, the filamentous group saw a decrease in taxonomic importance by -24% in the first period. The articulated calcareous group’s importance fell by 7%, while the leathery macrophyte group saw a 3% increase. The corticated foliose group’s taxonomic importance fluctuated, ending with a -4% decline in the final period.

#### Changes in functional group composition

The dissimilarity in functional group composition increased over time. From 1800 to 1927 to 1940–1978, dissimilarity was 30%, rising to 37% by 2010–2013. However, the dissimilarity between more recent periods was lower, showing smaller shifts in composition.

#### Functional equitability

The equitability of functional groups in the seaweed assemblage showed a linear decline, from 0.94 to 0.66 across time.

#### Functional diversity

The diversity of functional groups linearly declined from 1.04 to 0.73 over time.

### Functional strategy dynamics

#### Species richness

**Fig. 4:**
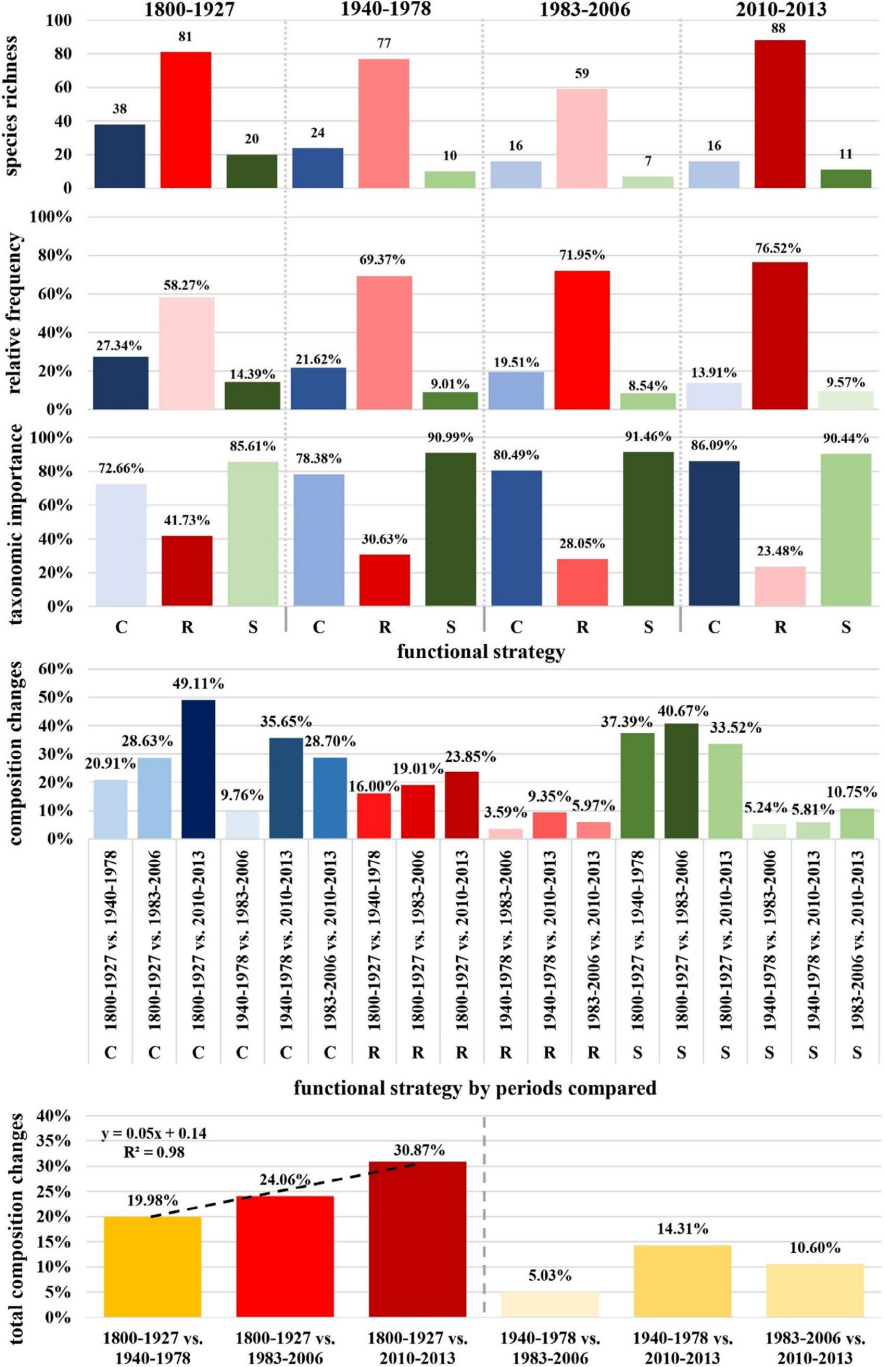
Functional strategy dynamics across 213 years of anthropogenic disturbance. Letters *C*, *R*, and *S* stand for competitive, ruderal, and stress-tolerant strategists.

Species richness within functional strategies declined overall between 1800 and 1927 and 1983–2006, with the competitive (C) strategy dropping from 38 to 16 species, the ruderal (R) strategy decreasing from 81 to 59, and the stress-tolerant (S) strategy declining from 20 to 7 (Fig. 4). However, from 2010 to 2013, R-strategy species recovered to 88 species, while C- and S-strategies remained stable at 16 and 11 species, respectively, indicating some recovery for R-strategists but continued low richness for C- and S-strategists.

#### Relative frequency

The R-strategy consistently increased in relative frequency from 58 to 76% over time, dominating the seaweed assemblage. C-strategy frequency declined from 27 to 14%, while S-strategy remained the least frequent, ranging from 14 to 10%.

#### Taxonomic importance within functional strategies

C-strategists maintained high taxonomic importance (73–86%) across periods, despite declining species richness. R-strategists’ taxonomic importance steadily declined from 42 to 23%, even as their frequency increased, raising redundancy. S-strategists maintained the highest taxonomic importance (86–91%), showing very low redundancy.

#### Competitive strategists, period by period

The Jaccard dissimilarity index for C-strategists increased significantly over time, from 21% in 1800–1927 vs. 1940–1978 to 49% in 1800–1927 vs. 2010–2013. More recent periods (1940–1978 vs. 1983–2006) showed lower dissimilarity (10%), suggesting smaller changes in C-strategy composition in the later phases of the time series.

#### Ruderal strategists, period by period

R-strategists displayed lower dissimilarity compared to C-strategists, with indices ranging from 16% (1800–1927 vs. 1940–1978) to 24% (1800–1927 vs. 2010–2013). Recent periods exhibited minimal change, with dissimilarity dropping to 4% (1940–1978 vs. 1983–2006), indicating greater stability in R-strategy composition.

#### Stress-tolerant strategists, period by period

S-strategists initially showed high dissimilarity, with 37% (1800–1927 vs. 1940–1978), but this gradually decreased to 34% (1800–1927 vs. 2010–2013). In recent periods, dissimilarity remained low, around 5%, suggesting a stable S-strategy composition after initial changes.

#### Total changes in functional strategy composition, period by period

The dissimilarity between 1800 and 1927 and 1940–1978 was 2%, increasing to 30% between 1800 and 1927 and 2010–2013, showing the largest change.

### C/SR index metric: quantitatively assessing functional change

The C/SR ratio showed a linear trend of decrease over the studied periods. In 1800–1927, the ratio was 38%, indicating that the C-strategy was less frequent than the combined frequency of S- and R-strategies but still constituted a significant proportion of the total seaweed assemblage.

**Fig. 5:**
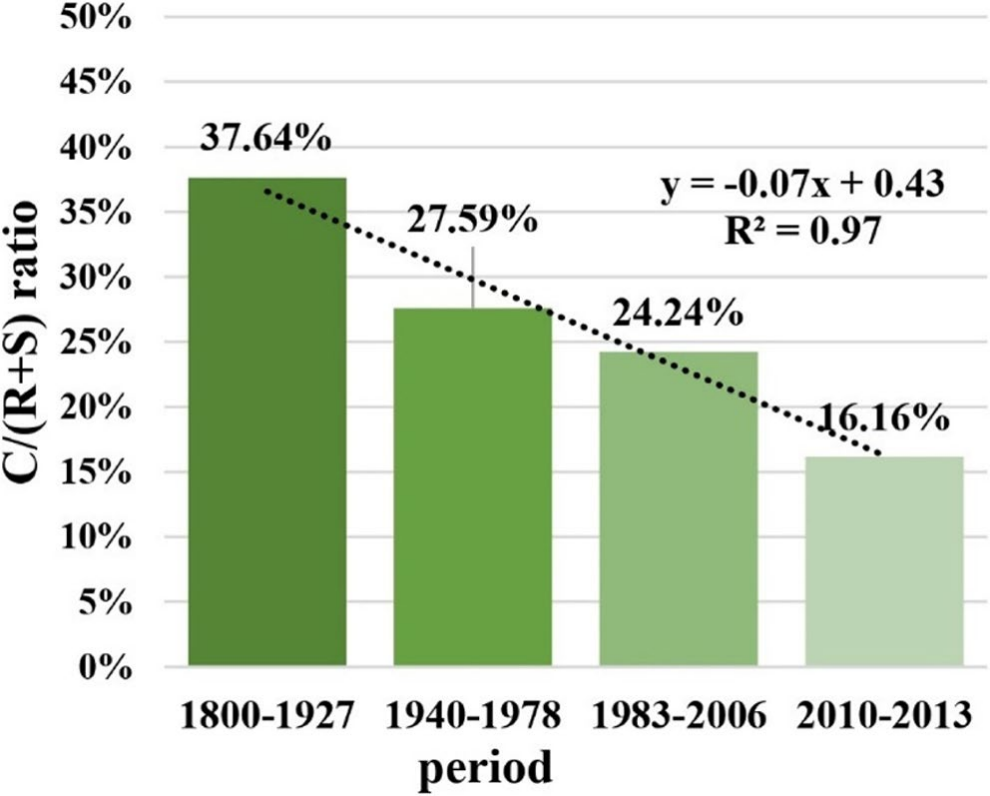
C/SR ratio index: a new index to quantitatively track and predict changes in functional strategy assembly composition. Letters stand for functional strategies. C, competitive strategy frequency. R, ruderal strategy frequency. S, stress-tolerant strategy frequency.

By 1940–1978, the C/SR ratio had decreased to 28%, reflecting a further decline in the relative frequency of C-strategy compared to S- and R-. This trend continued into 1983–2006, dropping the ratio to 24%. In the most recent period, the C/SR ratio reached its lowest point at 16% (Fig. 5).

## Discussion

### What changes have occurred across the seaweed taxa?

The seaweed assemblage of Rio de Janeiro Bay has undergone substantial taxonomic shifts, characterized by a marked decline in species and genus richness from the 19th to late 20th century, followed by a modest recovery in recent years. Records from 1800 to 1927 establish a historical baseline of 139 species, representing the bay’s biodiversity before significant anthropogenic pressures. By 1940–1978, species richness had dropped to 111, a loss likely driven by intensifying urbanization and industrial activity^2,4^, further increased in the following 1983–2006 period, of only 82 species. However, by 2010–2013, a small recovery occurred, with species richness rising to 115. Despite this rebound, the long-term trend remains one of declining biodiversity, with a turnover of -17% from the historical baseline to the present times.

The increase in richness may be attributable to new species invasions^5^, enhanced taxonomic resolution^23,24,32^, or more comprehensive sampling methods^33^. These factors, combined with heavy international maritime traffic from the bay’s many harbors^34,35^, highlight the ongoing challenge of assessing native biodiversity patterns amidst the potential introduction of exotic species^35^.

The recent rise in species richness warrants further long-term monitoring to determine if it marks the onset of a new ecological equilibrium or is merely a transient oscillation^36,37^. Unraveling the drivers of this pattern will be crucial for informing conservation strategies and ensuring the sustainable management of Rio de Janeiro’s coastal biodiversity^38,39^.

### How have functional groups and strategies changed?

Human-induced changes across Rio de Janeiro Bay over two centuries saw an increase in filamentous and crustose algae, alongside declines and local extinctions in canopy and subcanopy-forming seaweeds, such as leathery macrophytes (such as *Sargassum* – locally extinct), articulated calcareous algae (e.g., *Halimeda*, *Penicillus*, *Corallina* and several *Jania* species – locally extinct) and corticated foliose algae (e.g., *Canistrocarpus*, *Dictyota*, *Lobophora* and *Spatoglossum* – locally extinct). Filamentous algae species increased by 62%, and crustose algae by 8%. In contrast, 54% of corticated macrophytes, 29% of leathery macrophytes, 21% of articulated calcareous algae, and 17% of sheet (or foliose) algae became locally extinct. Thus, the overall decline in species and genus richness is strongly influenced by functional group identity^40,41^. This pattern mirrors the global decline of kelp and canopy-forming seaweeds, giving way to filamentous algae dominance^40,42–45^.

The functional group composition shift from bigger, morphologically and anatomically complex algae to smaller and simpler forms has likely impacted the benthic structural complexity and spatial heterogeneity needed for high marine biodiversity^46–50^. Structural simplification brought about by disturbance and stressor effects is well supported by literature on seaweed functional ecology^19,27,51–53^ and surveys^43,50,54^. The seaweed canopy to turf phase shift is more clearly illustrated by the linear decline in both functional equitability and functional diversity, paired with the improvement in filamentous and ruderal algae metrics at the expense of other algal groups and strategies. This underscores the historical transition from a *Sargassum* kelp forest baseline state to a depauperate and functionally homogenized one, increasingly dominated by filamentous and crustose algae, on par with global trends of functional change in seaweed-structured benthos worldwide^44,48,50,55,56^. The decline in competitive strategy metrics compared to ruderals’ also highlights an environmental shift in structuring drivers, with competition-driven structuring diminishing further towards heightened disturbance and stressor structuring^28,30,45,57,58^, from a 37.64% C/SR index baseline to a 16.16% C/SR index nowadays.

### Has anthropization favored one evolutionary strategy over another?

The ruderal strategy was overwhelmingly favored, showing benefits across all metrics. Competitive strategists saw the greatest decline in all metrics except in taxonomic importance, the opposite of the pattern found for urbanization effects on land plant community assembly^59^. Lowered richness also necessarily lowered the high interspecific competition required for competitive strategist dominance^28,30^.

Surprisingly, stress-tolerant strategists saw mixed responses from anthropization, remaining with stable metrics. This is contrary to what was expected given the rise in new anthropogenic stresses^60–63^. We speculate that the minor decline in stress-tolerant seaweeds may reflect the overall retention of previous patch occupancy^64–66^, while previously competition-structured patches, once dominated by competitive strategists, now became disturbance-structured patches, dominated by ruderal algae. We further speculate that anthropogenic disruptions have therefore likely created a patch mosaic too rapidly changing^67^ for competitive and stress-tolerant strategists to thrive.

### C/SR ratio index: quantitatively tracking functional changes in rocky shores and reefs with seaweed dominance

The C/SR index is designed to quantify and track baseline conditions and functional changes. This allows for a quantitative evaluation of conservation and restoration efforts by determining the significance of changes relative to predicted functions. Our index provided a clear, linear metric for assessing functional changes across a two-century historical series. It offered a consistent measure that was less affected by species-level dynamics compared to taxonomic and functional group analyses. The latter could misleadingly suggest environmental improvements in Rio de Janeiro Bay, of which the opposite happened^5–10^, based on increased species and genus richness from 1983 to 2006 to 2010–2013.

Considering the limitations of current functional group approaches, as highlighted in our recent review^19^, a shift towards using functional strategy metrics could offer a more generalizable alternative for assemblage-wide assessment. Rather than continuing the trend of increasing parameter numbers in trait-based models, which has been prevalent for seaweeds^[e.g.,68,69^, land plants^70,71^, and trait-based ecology in general^72^, functional strategy metrics could offer a more streamlined and effective framework for assemblage-level analyses^73^, especially useful for time- and resource-limited conservation efforts.

## Conclusion

The synthesis of the three levels of analysis, taxonomy, functional groups, and functional strategies, corroborated synergistically to the characterization of an evolving picture of a seaweed assemblage subjected to centuries of increasing human disturbance. From the taxonomic data, we saw a gradual reduction and turnover in the bay’s biodiversity. From the functional group data, we saw the decline of morphologically complex and canopy-forming forms in favor of simpler and smaller seaweed forms. From the functional strategy data, we saw the historical decline of competitive-structuring as an assemblage driver in favor of an increasingly disturbed (ruderal-favoring) and stressful (stress-tolerant-favoring) environment. The new monitoring index we proposed captured in a single formula this gradual phytobenthic restructuring, with a very high explanatory power (R^2^ = 0.97).

## Electronic supplementary material

Below is the link to the electronic supplementary material.

Supplementary Material 1

Supplementary Material 2

## Data Availability

All data relevant to this study are provided within the manuscript and its supplementary materials.
