# Supplementary material for: Seaweed functional strategies, functional groups, and taxon dynamics through a 213-year historical series of Rio De Janeiro Bay
Source: Sci Rep. 2024 Oct 29;14:25932. doi: 10.1038/s41598-024-77284-y (PMC11522484; doi:10.1038/s41598-024-77284-y)
Supplement: Supplementary file 2 — Supplementary Material 2 [file 41598_2024_77284_MOESM2_ESM.pdf]

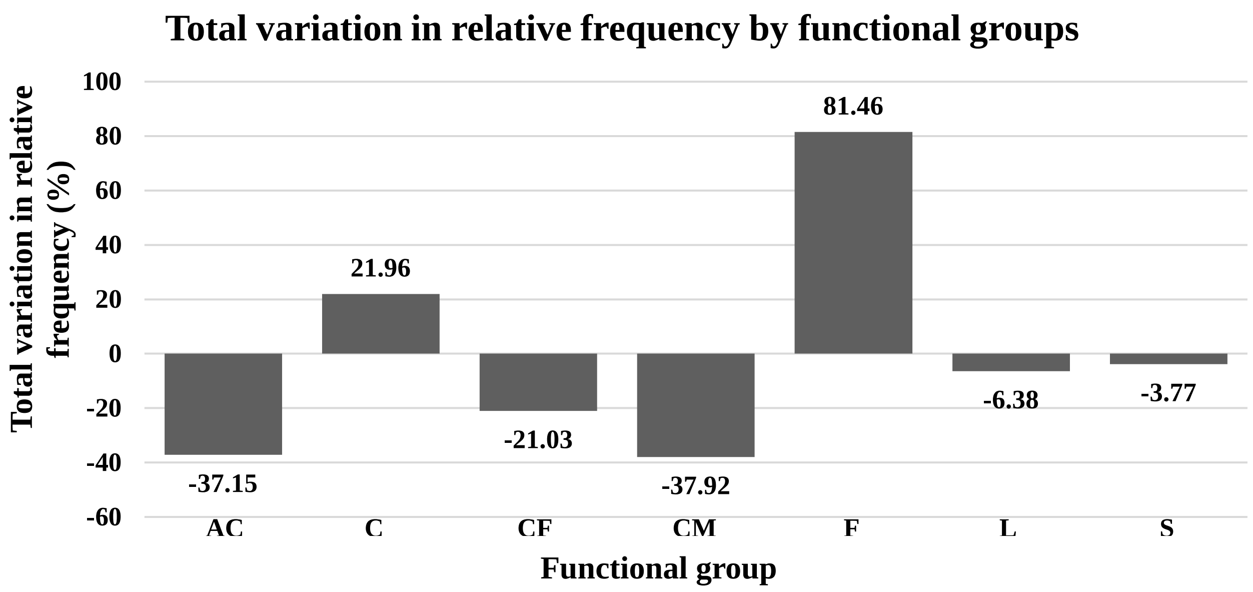

Supplementary Figure 1. Total variation in the relative frequency of each seaweed functional group. AC: Articulated Calcareous. C: Crustose. CF: Corticated Foliose. CM: Corticated Macrophyte. F: Filamentous. L: Leathery Macrophyte. S: Sheet (Foliose).
